# Factors related to the patient safety climate in an emergency hospital*

**DOI:** 10.1590/1518-8345.3353.3273

**Published:** 2020-06-01

**Authors:** Dayse Edwiges Carvalho Castilho, Ana Elisa Bauer de Camargo Silva, Fernanda Raphael Escobar Gimenes, Ranielle de Lima Silva Nunes, Ana Claudia Andrade Cordeiro Pires, Cristina Alves Bernardes

**Affiliations:** 1 Escola de Saúde Pública Cândido Santiago, Educação Profissional em Saúde, Goiânia, GO, Brazil.; 2 Universidade Federal de Goiás, Faculdade de Enfermagem, Goiânia, GO, Brazil.; 3 Universidade de São Paulo, Escola de Enfermagem de Ribeirão Preto, PAHO/WHO Collaborating Centre at the Nursing Research Development, Ribeirão Preto, SP, Brazil.; 4 Hospital das Clínicas, Unidade de Terapia Intensiva, Goiânia, GO, Brazil.; 5 Universidade Federal de Goiás, Núcleo de Estudos em Saúde Coletiva, Goiânia, GO, Brazil.; 6 Hospital Materno Infantil, CCIH, Goiânia, Goiás, Brazil.; 7 Hospital do Coração Anis Rassi, Qualidade, Goiânia, GO, Brazil.

**Keywords:** Patient Safety, Organizational Culture, Safety Management, Nursing, Quality of Health Care, Emergency Service, Hospital, Segurança do Paciente, Cultura Organizacional, Gestão da Segurança, Enfermagem, Qualidade da Assistência à Saúde, Serviço Hospitalar de Emergência, Seguridad del Paciente, Cultura Organizacional, Gestión de la Seguridad, Enfermería, Calidad de la Atención de Salud, Servicio de Urgencia en Hospital

## Abstract

**Objective:**

to verify the relationship between the socio-demographic and work profile of the nursing professionals and the patient safety climate in a public emergency hospital.

**Method:**

a cross-sectional study carried out with 177 nursing professionals from a public emergency hospital. For data collection, the Safety Attitudes Questionnaire - Short Form 2006 was used, validated and cross-culturally adapted to the Portuguese language. To check the factors related to the instrument’s domains, bivariate and multivariate analyses were performed.

**Results:**

working in the medical and surgical clinic or emergency room, on a night shift, and having the intention to leave nursing, reduced the general safety climate in the multiple regression analysis. The younger professionals, with less than four years in the institution, and those who worked in the night shift had a lower safety climate related to the perception of the management. On the other hand, having a work contract with a hired worker improved the general safety climate and workplace satisfaction.

**Conclusion:**

identifying predictors on patient safety scores is an important management tool that allows diagnosing, planning and executing activities from the domains that need to be improved.

## Abstract (pt)

**Objetivo:**

verificar a relação entre o perfil sociodemográfico e laboral dos profissionais de enfermagem e o clima de segurança do paciente de um hospital público de urgências.

**Método:**

estudo transversal realizado com 177 profissionais de enfermagem de um hospital público de urgências. Para a coleta de dados, foi utilizado o *Safety Attitudes Questionnaire* - *Short Form* 2006, validado e adaptado transculturalmente para a língua portuguesa. Para verificar os fatores relacionados aos domínios do instrumento, foram realizadas análises bivariadas e multivariáveis.

**Resultados:**

atuar na clínica médica e cirúrgica ou pronto-socorro, em turno noturno e possuir intenção de sair da enfermagem diminuiu o clima de segurança geral, em análise de regressão múltipla. Os profissionais mais novos, com menos de quatro anos na instituição e os que atuavam no turno noturno apresentaram menor clima de segurança relacionado à percepção da gerência. Em contrapartida, ter vínculo de trabalho celetista melhorou o clima de segurança geral e a satisfação no trabalho.

**Conclusão:**

identificar preditores sobre os escores de segurança do paciente é uma importante ferramenta gerencial que permite diagnosticar, planejar e executar atividades a partir dos domínios que precisam ser aprimorados.

## Abstract (es)

**Objetivo:**

verificar la relación entre el perfil sociodemográfico y laboral de los profesionales de enfermería y el clima de seguridad del paciente en un hospital público de urgencias.

**Método:**

estudio transversal realizado con 177 profesionales de enfermería de un hospital público de urgencias. Para la recopilación de datos, se utilizó el *Safety Attitudes Questionnaire* - Short *Form* 2006, validado y adaptado transculturalmente al idioma portugués. Para verificar los factores relacionados con los dominios del instrumento se realizaron análisis bivariados y multivariados.

**Resultados:**

actuar en la clínica médica y quirúrgica o en la sala de emergencias, en un turno nocturno, y tener la intención de abandonar la enfermería, redujo el clima de seguridad general en el análisis de regresión múltiple. Los profesionales más jóvenes, con menos de cuatro años en la institución, y aquellos que trabajaban en el turno nocturno tuvieron un clima menor de seguridad relacionado con la percepción de la gerencia. Por otro lado, tener un contrato de trabajo con un trabajador contratado mejoró el clima general de seguridad y la satisfacción laboral.

**Conclusión:**

identificar los predictores en las puntuaciones de seguridad del paciente es una herramienta de gestión importante que permite diagnosticar, planificar y ejecutar actividades desde los dominios que deben mejorarse.

## Introduction

Worldwide, 64 million disability-adjusted life years are lost annually due to unsafe care^(^ 1 ^)^. This means that adverse events, defined as an incident that resulted in harm to the patient^(^ 2 ^)^ are, probably, one of the top ten causes of harm and death^(^ 1 ^)^.

In Brazil, the bulletin released by the National Health Surveillance Agency (*Agência Nacional de Vigilância Sanitária*, ANVISA - abbreviation in Portuguese), in 2017^(^ 3 ^)^, revealed that most safety incidents (defined as an event or a circumstance that could have resulted in, or resulted in, unnecessary harm to the patient)^(^ 2 ^)^, reported to the National Health Surveillance System, occurred in hospitals (94%) and in exclusive urgent and emergency services (2.3%). When considering the number of health care-related incidents reported per hospital unit, urgent and emergency services ranked third (7.6%)^(^ 3 ^)^.

In urgent and emergency services, stress situations related to poor working conditions, inadequate sizing of the nursing team, limited resources, overcrowding, the long waits and the exposure of workers to stress that can impact the quality and safety of care^(^ 4 ^)^. In these institutions, the main risks to patient safety are related to the following: clinical management, diagnosis and medication errors^(^ 5 ^)^, adverse events resulting from insertion, handling and the maintenance of medical devices (such as cannulas, venous catheters, tubes and drains), bronchoaspiration and trauma^(^ 6 ^)^.

It is also observed that there is a greater number of risk behaviors that impact the mortality rate^(^ 7 ^)^ in a situation of overcrowding. It is important to note that, although urgency and emergency services have a high flow of patients and complex demands, there is no justification for risking patient safety^(^ 8 ^)^.

Patient safety is considered a priority attribute of the quality of the health systems^(^ 9 ^)^, a fundamental principle of patient care and a critical component of management^(^ 10 ^)^. For care to be considered of quality, it is necessary that it is safe, effective, timely, efficient, equitable and patient-centered^(^ 11 ^)^. In addition, patient-centered care, teamwork and the safety climate are related to better patient outcomes, to the greater number of safety incident notifications and to lower rates of adverse events, death and hospital readmission^(^ 12 ^)^.

The patient safety climate is also closely related to the safety culture in that it represents people’s perceptions and organizational practices that reflect basic assumptions and beliefs based on the culture^(^ 13 ^)^. The safety climate plays an important role in reducing safety incidents^(^ 14 ^)^. For example, medication errors are less frequent, and rates of hospital readmission are lower in units where the health team classifies the safety climate as positive^(^ 13 ^)^. A positive safety climate improves not only productivity and interpersonal relationships^(^ 14 ^)^ but also the quality of nursing care^(^ 15 ^)^.

There are several factors that influence the patient safety climate^(^ 16 ^-^ 18 ^)^. The following can be mentioned: the perception of the professionals regarding the management, that is, the degree of approval of the teams in relation to the managerial actions; team work that addresses the quality of collaboration among the staff; workplace satisfaction, which is the degree to which people feel positive in their work experience and working conditions, defined by the quality of the work environment and logistical support^(^ 19 ^)^. In addition, the workload, professional exhaustion and the intention to leave the workplace affect the quality of care provided to patients by the nursing professionals^(^ 20 ^)^.

Investigations carried out in Brazil have shown an atmosphere of patient safety that is not satisfactory and with fragility in the perception of stress, in the perception of management and in the working conditions. Regarding the positive points observed in the patient safety culture, researchers find workplace satisfaction as a positive dimension^(^ 21 ^-^ 23 ^)^.

In another study that aimed to assess the climate of patient safety in a Brazilian urgency and emergency unit, the researchers identified an unfavorable climate, especially with regard to management actions in patient safety management^(^ 24 ^)^.

Measuring the safety climate can be useful in diagnosing, planning and executing activities based on the domains that need to be improved and the intrinsic and extrinsic factors of the professionals who need attention. Furthermore, it is important to measure the patient safety climate in different units of the same service, because it may differ from the general climate of the institution. This diagnosis will allow for the identification of opportunities for improvement^(^ 25 ^)^.

Most studies investigated the safety climate in a single unit and focused on locations with high intrinsic risk, such as operating rooms^(^ 26 ^)^ and intensive care units^(^ 27 ^-^ 28 ^)^. However, studies aimed at measuring the safety climate in urgent and emergency services are still scarce.

It should also be noted that the safety climate may differ among people working in the same unit and/or department^(^ 29 ^-^ 30 ^)^, and it is important to investigate this relationship in order to direct strategies aimed at improving the safety climate both at the organizational and individual levels. According to a research study conducted in two manufacturing industries in Malaysia, personal factors, such as the employees’ personality traits, age, gender, income and educational level are examples that can contribute to the adoption of risky behaviors and to the worsening of the safety climate^(^ 29 ^)^. However, this relationship has not yet been well established in health institutions, particularly among the nursing professionals^(^ 31 ^)^.

In this context, identifying the factors that are impacting the patient safety climate is an important tool to improve the quality of care, as it makes it possible to diagnose areas that need improvement within health institutions and among the nursing professionals^(^ 32 ^)^.

This study aimed to verify the relationship between the socio-demographic and work profile of the nursing professionals and the patient safety climate in a public emergency hospital.

## Method

This is a cross-sectional analytical study carried out in a public emergency hospital located in Aparecida de Goiânia, Goiás, Brazil, from February to August 2017. The institution is managed by a Social Organization and has 100 beds, of which 62 are for medical and surgical clinic, 23 are for the Emergency Room (ER), 10 for the Adult Intensive Care Unit (ICU), one for Hemodialysis and four for the Post-Anesthetic Recovery Room (PACU) of the Surgical Center Unit (SCU). The monthly mean number of patients is 2,700 and the mean numbers of hospitalizations/month in 2017 were 244 in the ER, 240 in the medical and surgical clinic, and 5 in the ICU. As it is an emergency hospital, patient turnover is high and the mean stay is low, varying around 2.8 days.

The population consisted of 316 nursing professionals who could work in patient care, in related sectors, or had a nursing coordination position. Those who were absent from work during the period of data collection due to vacation or leave, of any kind, were excluded, thus making an eligible population of 276 professionals. Losses were considered as participants who, after three attempted approaches, did not answer the questionnaires or answered less than 25% of the questionnaires’ questions. Thus, 177 professionals agreed to participate in the research and met the inclusion criteria.

The Safety Attitudes Questionnaire - SAQ - Short Form 2006 was used, validated and cross-culturally adapted to the Portuguese language^(^ 33 ^)^. The self-responsive instrument has good psychometric properties and reliability. It is divided into two parts. The first has five questions regarding gender, position, time in the specialty, and main role. The second is composed of 41 items that encompass six domains: teamwork climate, safety and workplace satisfaction climate, perception of stress, perception of the unit and hospital management and working conditions. Items 14, 33, 34, 35 and 36 do not belong to any domain, although they are considered in the final score of the instrument.

The participants assign a score to each statement contained in the instrument, based on a 5-point Likert bipolar scale: strongly disagree (0 points), slightly disagree (25 points), neutral (50 points), slightly agree (75 points) and strongly agree (100 points). Each statement, also, presents the “does not apply” answer option, which is not computed in the calculation of the domain score. Therefore, the scores are counted as follows: first, the reverse items corresponding to questions 2, 11 and 36 of the SAQ. Then, the questions of the instrument are ordered in domains that contain a set of items whose scores are added up. The final result of the sum of the scores is divided by the number of items in the domain. A positive overall climate score is considered to be a general final score greater than or equal to 75^(^ 33 ^-^ 34 ^)^.

To complement socio-demographic and working data, a form constructed by the researchers was applied. The intention to leave the workplace was measured using a 4 points Likert scale (none = 1 point; low = 2 points; moderate = 3 points; high = 4 points).

Data collection took place upon acceptance by the nursing professional and signature of the Free and Informed Consent Form (FICF). For data collection, each participant was approached individually at their location and during their working hours. The participant received the self-administered questionnaire to be filled out and, later, returned it to the researchers who were waiting on the spot.

The data were entered in duplicate in Microsoft Excel and analyzed using the Statistical Package for the Social Sciences (SPSS), version 24.0, and STATA, version 14.0. The normality of the quantitative variables (age, number of nursing professionals per shift, number of patients per professional, and SAQ domains) was verified using the Kolmogorov-Smirnov test with Lilliefors correction. Then, the descriptive analysis was performed. The quantitative variables were presented as median and Interquartile Range (IQR) due to the absence of normality for all the variables, except for the SAQ domain scores that were presented as mean and standard deviation (SD). The qualitative variables were presented as absolute and relative frequencies in the descriptive analysis of labor and sociodemographic characteristics.

The analysis of the internal consistency of the SAQ was performed by the standardized Cronbach Alpha coefficient, with good internal reliability > 0.7. The Intraclass Correlation Coefficient (ICC) was also used. Values below 0.5, between 0.5 and 0.75, between 0.76 and 0.90, and > 0.90 are indicative of poor, moderate, good, and excellent reliability, respectively.

To analyze the factors related to the scores of the SAQ domains, bivariate and multiple analyses were performed. Initially, in the bivariate analysis, simple linear regression analyses were performed between each independent variable (gender, age, type of bond, number of bonds, time working in the hospital, work sector, work shift, intention to leave the workplace, and intention to leave nursing) and the dependent variables analyzed (SAQ domains). Then, a significance level of 20% (p-value < 0.20) by the *t* test of the regression was used as a selection method for entering the variable in the model^(^ 35 ^)^. Thus, only variables with p < 0.20 were included in the multiple linear regression models. Finally, multiple linear regression models were performed, using the automatic stepwise method to adjust the variable confusion potentials of the models^(^ 36 ^)^. The statistical significance of multiple linear regressions was also established by the *t* test^(^ 37 ^)^. The results of the regression models were presented as an adjusted regression coefficient (ββ_aj_) and respective 95% confidence intervals (95% CI). In the present study, only the statistically significant variables of the multiple regression models were presented in the tables. Variables with a p-value < 0.05 in the multiple regression analysis were considered statistically significant.

Model 1 (general score of SAQ) was adjusted for the following independent variables: age, gender, number of bonds, sector of employment, type of bond, length of time in the hospital, work shift, intention to leave nursing and intention to leave the workplace; model 2 (climate related to team work) was adjusted for age, gender, work sector, intention to leave nursing and intention to leave the workplace; model 3 (perception of the unit and hospital management) was adjusted for age, gender, type of employment, sector of work, length of time in the hospital, work shift, intention to leave nursing and intention to leave the workplace; model 4 (safety climate) was adjusted for age, gender, work sector, type of employment, work shift, intention to leave nursing and intention to leave the workplace; model 5 (workplace satisfaction) was adjusted for age, gender, work sector, type of employment, work shift, intention to leave nursing and intention to leave the workplace; model 6 (working conditions) was adjusted for age, gender, work shift, sector of work, intention to leave nursing and intention to leave the workplace. Model 7 (stress perception) was adjusted for age, gender and intention to leave the workplace. It should be noted that only variables with a p-value < 0.20 were included in the regression models in the bivariate analysis.

This investigation is part of the project entitled “Safety climate among the multi-professional health team” approved by the Ethics Committee on Human and Animal Research of the Clinical Hospital, Federal University of Goiás, with opinion number 1.887.147 and CAAE: 49279115.4.0000.5078.

## Results

Of the nursing professionals, 177 accepted to participate in the study and met the inclusion criteria, generating a response rate of 64.1%. The p-value of the Kolmogorov-Smirnov normality test with Lilliefors correction showed an absence of normality for all the quantitative variables: age (p-value = 0.024), number of nursing professionals *per* shift (p-value < 0.001), number of patients per professional (p-value < 0.001), working conditions domain (p-value = 0.034), general domain (p-value = 0.011) and the remaining SAQ domains (p-valor < 0.001). Of the total, 27.1% were nurses and 72.9% were nursing technicians or assistants. Most respondents were female (85.9%) and the median age of the professionals was 40 years old (IQR: 33.0-45.5).

Of the professionals, 48.0% were statutory and 52.0% were hired officers; 53.7% reported having more than one workplace; 54.8% worked during the day shift, and 45.2% at night. Among the nursing technicians and assistants, 49.6% had more than 10 years of training while 14.7% underwent some type of complementary training. Among the nurses, 52.1% had 5 to 10 years of graduation and 93.8% had some complementary training.

The majority (54.3%) of the nursing technicians and assistants had worked in the hospital for more than five years while almost 1/3 of the nurses had been in the institution for less than a year. Among the professionals participating in the research, 17.0% had a moderate/high intention to leave the workplace and 8.5% had a moderate/high intention to leave nursing. The median number of nursing professionals *per* shift in the unit/sector was 6.0 professionals (IQR: 36.0-60.0). The median number of patients per professional was 10.0 (IQR: 3.5-15.0).

The mean of the overall SAQ score was 67.6 (SD: 14.2). The total instrument presented a Cronbach’s alpha of 0.899 and an ICC of 0.890, suggesting good consistency and internal reliability, respectively. The Cronbach’s alpha (α) and ICC scores for each domain were the following: teamwork climate (mean: 70.4; SD: 19.5; α = 0.602; ICC: 0.538); safety climate (mean: 60.3; SD: 18.4; α = 0.623; ICC: 0.597); work satisfaction (mean: 78.8; SD: 21.8; α = 0.805; ICC: 0.773); stress perception (mean: 71.8; SD: 26.3; α = 0.759; ICC: 0.756); perception of the unit and hospital management (mean: 58.9; SD: 19.4; α = 0.815; ICC: 0.804); and working conditions (mean: 65.4; SD: 23.6; α = 0.735; ICC: 0,735).

Tables 1 to 5 show the factors associated with the scores of the SAQ domains obtained in linear regression models, stepwise method. It should be noted that the perception of stress was not associated with any socio-demographic and work characteristics.

**Table 1:** Factors related to the general safety climate of the nursing professionals. Aparecida de Goiânia, GO, Brazil, 2017

Variables	β_aj_ *	CI 95%^†^	p-value
**Sector**			
SMC/SCU^‡^ (R^§^)			
ICU^ǁǁ^	-4.25	-10.23; 1.71	0.161
Medical and Surgical Clinic	-11.07	-16.89; -5.25	<0.001
Emergency Room	-11.30	-15.77; -6.83	<0.001
**Type of bond**			
Statutory (R^§^)			
Working under the consolidation of Labor Laws	7.00	1.85; 12.16	0.008
**Work shift**			
Day (R^§^)			
Night	-5.60	-9.45; -1.75	0.005
**Intention to leave nursing**			
None/Low (R^§^)			
Moderate/High	-8.27	-15.08; -1.46	0.018
F value (p-value): 7.80 (< 0.001)			
R^2^: 0.298			

* β = Regression coefficient;

† 95% CI = 95% Confidence Interval;

‡ SMC/SCU = Sterilization Material Central/Surgical Center Unit;

§ R = Reference category;

ǁǁ ICU = Intensive Care Unit

**Table 2:** Factors related to the perception of the unit and hospital management of the nursing professionals. Aparecida de Goiânia, GO, Brazil, 2017

Variables	β_aj_ *	CI 95%^†^	p-value
**Sector**			
SMC/SCU^‡^ (R^§^)			
ICU^ǁǁ^	-7.75	-17.06; 1.55	0.102
Medical and Surgical Clinic	-14.51	-22.61; -6.40	0.001
Emergency Room	-15.65	-22.81; -8.49	<0.001
**Working time in the hospital**			
< 6 months (R^§^)			
6-11 months	-14.54	-26.14; -2.94	0.014
1-4 years	-9.68	-17.07; -2.29	0.011
> 5 years	-9.37	-20.90; 2.15	0.110
**Work shift**			
Day (R^§^)			
Night	-10.36	-15.75; -4.99	<0.001
**Intention to leave nursing**			
None/Low (R^§^)			
Moderate/High	-9.96	-19.14; -0.79	0.033
F value (p-value): 7.37 (< 0.001)			
R^2^: 0.232			

* β = Regression coefficient;

† 95% CI = 95% Confidence Interval;

‡ SMC/SCU = Sterilization Material Central/Surgical Center Unit;

§ R = Reference category;

ǁǁ ICU = Intensive Care Unit

**Table 3:** Factors related to the safety climate of the nursing professionals. Aparecida de Goiânia, GO, Brazil, 2017

Variables	β_aj_ *	CI 95%^†^	p-value
**Sector**			
SMC/SCU^‡^ (R^§^)			
ICU^ǁǁ^	-4.23	-11.72; 3.25	0.266
Medical and Surgical Clinic	-10.65	-17.93; -3.37	0.004
Emergency Room	-11.42	-18.49; -4.35	0.002
**Type of bond**			
Statutory (R^§^)			
Working under the consolidation of Labor Laws	8.09	2.62; 13.55	0.004
**Work shift**			
Day (R^§^)			
Night	-9.83	-15.22; -4.43	< 0.001
F value (p-value): 6.06 (< 0.001)			
R^2^: 0.222			

* β = Regression coefficient;

† 95% CI = 95% Confidence Interval;

‡ SMC/SCU = Sterilization Material Central/Surgical Center Unit;

§ R = Reference category;

ǁǁ ICU = Intensive Care Unit

**Table 4:** Factors related to workplace satisfaction of the nursing professionals. Aparecida de Goiânia, GO, Brazil, 2017

Variables	β_aj_ *	CI 95%^†^	p-value
**Sector**			
SMC/SCU^‡^ (R^§^)			
ICU^ǁǁ^	-6.50	-14.26; 1.25	0.100
Medical and Surgical Clinic	-9.00	-17.61; -0.39	0.040
Emergency Room	-11.75	-18.78; -4.72	0.001
**Type of bond**			
Statutory (R^§^)			
Working under the consolidation of Labor Laws	14.38	7.47; 21.28	< 0.001
**Work shift**			
Day (R^§^)			
Night	-8.57	-14.86; -2.22	0.008
**Intention to leave the workplace**			
None/Low (R^§^)			
Moderate/High	-9.50	-18.29; -0.71	0.034
F value (p-value): 5.89 (< 0.001)			
R^2^: 0.274			

* β = Regression coefficient;

† 95% CI = 95% Confidence Interval;

‡ SMC/SCU = Sterilization Material Central/Surgical Center Unit;

§ R = Reference category;

ǁǁ ICU = Intensive Care Unit

**Table 5:** Factors related to the working conditions of the nursing professionals. Aparecida de Goiânia, GO, Brazil, 2017

Variáveis	β_aj_ *	IC 95%^†^	p-valor
**Setor**			
CME//UCC^‡^ (R^§^)			
UTI^ǁ^	-3,01	-14,46; 8,44	0,604
Clínica Médica e Cirúrgica	-22,82	-32,28; -13,35	< 0,001
Pronto-Socorro	-12,74	-21,51; -3,97	0,005
Valor de F (p-valor): 5,99 (< 0,001)			
R^2^: 0,215			

* β = Regression coefficient;

† 95% CI = 95% Confidence Interval;

‡ SMC/SCU = Sterilization Material Central/Surgical Center Unit;

§ R = Reference category; ^ǁǁ^ICU = Intensive Care Unit

In the regression analysis, it was found that acting in the ER reduced the general safety climate score by 11.30 points (β_aj_ = -11.30) (Table 1), by 11.71 points the score of the climate related to teamwork (β_aj_ = -11.71) (data not shown in the Tables), by 15.65 points the score of the perception of the unit and hospital management (β_aj_ = -15.65) (Table 2), by 11.42 points the security climate score (β_aj_ = -11.42) (Table 3), by 11.75 points the workplace satisfaction score (β_aj_ = -11.75) (Table 4), and by 12.74 points the domain score referring to the working conditions (β_aj_ = -12.74) (Table 5). This result indicates that acting in the ER contributed to the reduction of the general safety climate and of most of the domains.

Working in the medical and surgical clinic decreased the general safety climate score by 11.07 points (β_aj_ = -11.07) (Table 1), by 14.51 points the perception score management (β_aj_ = -14.51) (Table 2), by 10.65 points the safety climate score (β_aj_ = -10.65) (Table 3), by 9.00 points the score for workplace satisfaction (β_aj_ = -9.00) (Table 4), and by 22.82 points the score for the climate related to the working conditions (β_aj_ = -22.82) (Table 5). It was verified that the “working conditions” domain showed the greatest reduction related to working in the medical and surgical clinic when compared to the other domains.

For the general safety climate, the regression model showed an excellent fit (F = 7.80; p < 0.001) and explained 29.8% (adjusted R^2^: 0.298) of the variability of this variable in the study sample (Table 1).

A negative association was observed between scores of management perception and working time between 6 and 11 months (β_aj_ = 0.014), and 1-4 years (β_aj_ = 0.011). This suggests that the professionals with intermediate time in the hospital (6 months to 4 years) have less perception of management. For this domain, the regression model showed an excellent fit (F = 7.37; p < 0.001) and explained 23.2% (adjusted R2: 0.232) of the variability of the climate scores related to the perception of the unit and hospital management in the sample (Table 2).

It was verified that working in the night shift decreased the general safety climate score by 5.60 points (β_aj_ = -5.60) (Table 1), by 10.36 points the score of the climate related to the perception of the unit and hospital management (β_aj_ = -10.36) (Table 2), by 9.83 points the safety climate score (β_aj_ = -9.83) (Table 3), and by 8.57 points the climate score related to workplace satisfaction (β_aj_ = -8.57) (Table 4). For the “safety climate” domain, the regression model showed an excellent fit (F = 6.06; p < 0.001) and explained 22.2% (adjusted R2: 0.222) of the variability of this variable.

It was also verified that having a moderate/high intention to leave nursing reduced the overall climate score by 8.27 points (β_aj_ = -8.27) (Table 1) and by 9.96 points the management perception score (β_aj_=-9.96) (Table 2). The intention to leave the workplace contributed to the reduction of the workplace satisfaction score by 9.50 points (β_aj_ = -9.50) (Table 4). For the “working conditions” domain, the regression model also showed an excellent fit (F = 5.99; p < 0.001) and explained 21.5% (adjusted R2: 0.215) of the variability of this variable.

Hired professionals would better assess the safety climate and had better workplace satisfaction compared to statutory workers (β_aj_ = 8.09 and β_aj_ = 17.38, respectively) (Tables 3 and 4).

## Discussion

The fact that most of the nursing professionals are female and young adult converges with other national studies carried out with the nursing teams^(^ 38 ^-^ 40 ^)^. The reality of human resources with different work regimes, statutory and hired workers, was also found in another investigation^(^ 40 ^)^ and can be explained due to the movement of adoption of the management of public health services by social organizations, so that there are, within the same hospital, workers with different employment relationships and, consequently, with unequal rights and wage conditions^(^ 41 ^)^.

It was evident that almost all the nurses stated that they had some complementary training such as graduation, training and refresher courses. The participation of Nursing in permanent education programs results in changes in the work process and generates positive impacts on the care provided^(^ 42 ^)^ and, consequently, on patient safety.

The number of patients per professionals was close to double for nurses when compared to nursing technicians and assistants. In Brazil, the number of patients ranged from 9 to 27 *per* nurse and from 3 to 7 *per* assistant/technician, according to the inpatient units^(^ 43 ^)^. The increase in the workload is related to the occurrence of failures and health incidents^(^ 43 ^)^.

Four factors related to the general safety climate were identified: working in the ER or in the medical and surgical clinic, having the intention to leave the workplace or nursing, working in the night shift and having a working time greater than or equal to five years in the institution.

Working in the ER decreased the general patient safety climate, the atmosphere related to teamwork and the safety climate, in addition to decreasing workplace satisfaction, the atmosphere related to the perception of management and the climate associated with the working conditions. In an emergency service in a Brazilian hospital, the safety climate was negative, with a mean SAQ score below 75. The participants’ perceptions about the safety climate were negative, regardless of gender, length of service and position^(^ 24 ^)^.

The ER is characterized by being a unit with high patient turnover, which results in an increase in the workload and may justify the lower safety climate among these professionals. In addition, factors such as the severity and complexity of the patients can interfere with the safety climate of this sector. The manifestations of stress, tiredness, suffering, mental exhaustion and work overload of the nursing professionals who work in emergency units^(^ 4 ^)^ are also frequent.

An investigation into the influences on satisfaction, involvement at work, emotional exhaustion, the intention to leave work and psychosomatic suffering in nurses from emergency services showed that almost 20% of the respondents left work in less than 18 months. It also showed that work overload and high emotional demands can lead to a state of exhaustion. The authors concluded that high turnover is an important issue in the emergency services and that investments in collaborative and empathic leadership of supervisors are necessary, as well as the creation of a favorable working climate and opportunities for the professional growth of emergency nurses with the objective of stimulating their permanence at work^(^ 44 ^)^.

Urgency and emergency care units may present peculiar stressors such as meeting spontaneous demands, coping with unexpected situations, and work overload, in addition to problems related to work processes, infrastructure, and human and material resources^(^ 4 ^)^.

The professionals of the Medical and Surgical Clinic, characterized by the large volume of patients per month, also evaluated as worse the general safety climate, the safety climate, and the work satisfaction climate. They also negatively evaluated the management’s perception and working conditions when compared to the professionals in the ICU and SMC/SCU sectors.

The professionals with less than four years in the institution had a worse perception of the safety climate when compared to the management. In other studies, younger professionals also had less perception of the patient’s safety climate^(^ 17 ^,^ 40 ^)^. It is considered that the most recent professionals in the studied institution are mostly nurses, which can lead to a more critical view regarding the working conditions related to the training of new members, the supervision of nursing interns and the availability information for diagnostic and therapeutic decision-making.

Working at night reduced the safety climate of the general patient, the safety climate, and the climate related to the perception of the management. These data may indicate a probable distance between night shift professionals and their managers and leaders. Nurses working in the night shift showed an increase in the symptoms related to chronic and psychological fatigue, in addition to a greater intention to quit their workplace when compared to the others^(^ 45 ^)^.

Managers and leaders need to adopt strategies that enable the engagement, appreciation and participation of the collaborators in making organizational decisions. This measure can make employees feel part of the process of building the institution’s history and wish to remain in the workplace. This approach is particularly important for night shift professionals, who need to feel involved in the institution’s safety culture.

Researchers have demonstrated the relationship between reduced workplace satisfaction, increased stressors, and night shift work^(^ 46 ^-^ 47 ^)^. However, in this study, daytime professionals had less workplace satisfaction. This data may be related to a greater volume of activities planned for the day, such as scheduled exams and surgeries, hospital discharge, and patient transfer. The greater workload attributed to these professionals can result in decreased workplace satisfaction.

Having the intention to leave nursing contributed to the reduction of the general patient safety climate and the climate associated with the management’s perception. The intention to leave the workplace also reduced workplace satisfaction. The leadership styles and the lack of employer support among team members are very common reasons for the professionals to leave the workplace^(^ 47 ^)^ or to have the intention to leave the profession^(^ 48 ^)^.

Diverse research studies carried out with both nurses and nursing technicians and assistants demonstrated the direct correlation between the professional practice environment, the levels of emotional exhaustion, the perception of the quality of care, workplace satisfaction, and the intention to leave the workplace in the next 12 months. All of this can be influenced by the level of complexity and the management method of the institution^(^ 15 ^,^ 49 ^)^. In another study, the professionals with the intention to leave nursing had less perception of management and less workplace satisfaction^(^ 50 ^)^. Stress and lack of workplace satisfaction contribute to an increase in the turnover rate and to the intention to leave the profession^(^ 48 ^)^.

The nurses with the worst relations with doctors, with little autonomy and with low control over the environment, had a higher level of emotional exhaustion. This can negatively influence their perception of the quality of care, their satisfaction with work and their intention to leave the workplace^(^ 15 ^)^.

The hired professionals rated the safety climate better and had better workplace satisfaction. In another investigation, being statutory was negatively related (p < 0.05) to the perception of the management of the unit and of the hospital. The authors suggested that the professionals without workplace stability may present more positive responses, regarding the safety culture, because they fear retaliation in the workplace^(^ 17 ^)^. However, the study carried out in three public Brazilian urgency and emergency hospitals suggested that workplace stability can be a motivating strategy to guarantee the permanence of the professionals working in emergencies^(^ 51 ^)^.

The urgency and emergency units may have adverse working conditions that, negatively, affect the patient safety climate. For this reason, it is necessary to implement strategies to reduce stress, improve teamwork, and promote a safety culture for these workers.

The limitation of this work refers to its performance only with nursing professionals, which may limit the extrapolation of data to other scenarios and other professional categories. It is suggested to continue research in other health institutions, involving multi-professional health teams to survey factors that predict the patient safety climate.

## Conclusion

In the multiple regression analysis, working in the sectors of medical and surgical clinic (β_aj_ = -11.07), working in the emergency room (β_aj_ = -11.30), on a night shift (β_aj_ = -5.60), and having the intention to leave nursing (β_aj_ = -8.27) reduced the general safety climate. The youngest professionals (β_aj_ = 0.014), with less than four years in the institution (β_aj_ = 0.011), and those who worked in the night shift (β_aj_ = -10.36) had a lower safety climate related to the perception of the management. On the other hand, having a work contract with a hired worker improved the general safety climate (β_aj_ = 7.00) and workplace satisfaction (and β_aj_ = 17.38).

Understanding the factors related to the patient safety climate in nursing is of paramount importance, since these professionals are in a strategic situation to act in the prevention of the occurrence of adverse events and, when they occur, they must act accordingly in order to institute conducts to minimize the damage caused to the patient. Considering the context of urgency and emergency units, the proper performance of the nursing professionals becomes vital for the promotion of patient safety.

The evidence from this research may assist health managers in planning actions aimed at three aspects: investing in the valorization of the workers, stimulating dialog and promoting organizational learning based on mistakes. They can also contribute to the education of the nursing professionals, in order to promote reflection on organizational and personal issues that interfere in the provision of safe care. The findings of this study may also support future investigations aimed at assessing the patient safety climate in the health institutions. These efforts contribute to the improvement of the clinical practice in nursing and, consequently, to the dissemination and strengthening of the culture of patient safety in the country.
